# Development of a β-glucosidase hyperproducing mutant by combined chemical and UV mutagenesis

**DOI:** 10.1007/s13205-012-0095-z

**Published:** 2012-10-06

**Authors:** Ruchi Agrawal, Alok Satlewal, Ashok Kumar Verma

**Affiliations:** 1 Department of Biochemistry, College of Basic Sciences and Humanities, Govind Ballabh Pant University of Agriculture and Technology, Pantnagar, U.S. Nagar, 263145 Uttarakhand India; 2 Department of Microbiology, College of Basic Sciences and Humanities, Govind Ballabh Pant University of Agriculture and Technology, Pantnagar, U.S. Nagar, 263145 Uttarakhand India; 3 Present Address: Department of Bioenergy, DBT-IOC Centre, Sector 13, Faridabad, 121007 Haryana India

**Keywords:** β-Glucosidase, *Bacillus subtilis*, EMS mutagenesis, UV mutagenesis, SDS-PAGE, Size-exclusion chromatography

## Abstract

The extracellular β-glucosidase from microorganisms is generally produced in low levels. Therefore, in this study, a β-glucosidase hyperproducing mutant was developed by multiple exposures of ethyl methyl sulfonate (EMS) and ultraviolet (UV) radiation (both individually and jointly) to *Bacillus subtilis* strain (PS). The developed mutants were screened, selected and characterized. The mutant, PS-UM1 developed after UV exposure alone, indicated a small increase in β-glucosidase production (718 U/l) in comparison to the wild-type strain, PS (675 U/l). The mutant, PS-CM5 developed after EMS exposure alone, displayed a slightly better production (762 U/l) than both the above strains. However, after exposure of the wild-type strain to both UV and EMS mutagens jointly, a better mutant (PS-CM5-UM3) was developed with 1.2-fold increase in production (806 U/l). Further, optimization of culture conditions by classical “one-variable-at-a-time” approach was done to determine the optimum, pH, temperature and nitrogen sources. The selected mutant (PS-CM5-UM3) produced up to 1,797 U/l enzyme and was found to be stable for ten generations. The β-glucosidase from the selected mutant (PS-CM5-UM3) was concentrated and purified using ammonium sulfate, dialysis and size-exclusion chromatography. The enzyme displayed maximal activity at 60 °C and it was found to be fairly stable at temperatures up to 70 °C for 30 min. Its molecular weight was determined to be around 60 kDa by sodium dodecyl sulfate-polyacrylamide gel electrophoresis (SDS-PAGE).

## Introduction

β-Glucosidase (EC 3.2.1.21) together with endoglucanase (EC 3.2.1.4) and exoglucanase (EC 3.2.1.74) form a cellulase complex. All three enzymes work in a synergistic manner and hydrolyze the pretreated ligno-cellulosic biomass into glucose. β-Glucosidase catalyzes the final step in the cellulose hydrolysis, i.e., the breakdown of cellobiose into two glucose molecules. Thus, it plays an important role in improving the overall rate of cellulose degradation by removing the end product inhibition by cellobiose. β-Glucosidase catalyzes the hydrolysis of alkyl and aryl-β-glucosides, as well as diglucosides and oligosaccharides (Hoh et al. 1992; Mathew et al. 2008; Chen et al. 2012). β-Glucosidase is widely being used in biofuels, food, textile, leather, pulp and paper industries. It prevents the discoloration of fruit juices (Martino et al. 1994) and helps in the enzymatic release of aromatic compounds from glucosidic precursors present in fruits and fermenting products (Shoseyov et al. 1990; Guegen et al. 1996). Many natural flavor compounds such as monoterpenols, C-13 norisoprenoids and shikimate-derived compounds accumulate in fruits as flavorless precursors linked to mono or di-glycosides and require enzymatic or acidic hydrolysis for the liberation of their fragrances (Vasserot et al. 1995; Winterhalter and Skouroumounis 1997).

β-Glucosidase can be isolated from different sources such as plants, animals and microorganisms. Much research has been carried out on β-glucosidase of fungal origin (Mendels et al. 1971; Bailey and Nevalainene 1981; Sheir-Neiss and Montenecourt 1984; Gokhale et al. 1988; Durand et al. 1988; Rajoka et al. 1998, 2003, 2004; Chand et al. 2005; Dillon et al. 2006; Adsul et al. 2007; Chandra et al. 2009; Jun et al. 2009), but very less attention has been paid to bacterial sources (Zhou et al. 2008; Rashid and Siddiqui 1998). The production level of β-glucosidase in both fungal and bacterial culture filtrates is usually very low to be of any practical use (Lynd et al. 2002). Therefore, hyperproduction of β-glucosidase with better thermotolerant properties is required for industrial applications (Maki et al. 2009).

In this study, a selected *Bacillus subtilis* strain (PS) was improved with both physical (ultraviolet radiation) and chemical (ethyl methane sulfonate) mutagenesis. The developed mutants were screened and evaluated for enzyme production level. The β-glucosidase produced from a selected mutant was concentrated, purified and charecterized.

## Materials and methods

### Chemicals

Ethyl methyl sulfonate (EMS), p-nitrophenol β-d-glucopyranoside (pNPG), Luria–Bertani (LB) broth, Nutrient agar and other chemicals were procured from HiMedia Lab. Pvt. Ltd., Mumbai, India.

### Microorganism and culture conditions

The *B.subtilis* strain PS, used in this study, was originally isolated from sugarcane bagasse (Rawat 2009). The LB broth was used to grow the bacterial strain. It was maintained as 2 ml glycerol stocks in cryovials and kept at −20 °C for long-term storage.

### Mutagenesis and screening of mutants

A fresh active culture of *B. subtilis* strain PS was grown in LB media after overnight incubation at 37 °C from a glycerol stock. This strain was further cultured in 20 ml minimal media (containing K_2_HPO_4_ (5.8 g), KH_2_PO_4_ (4.5 g), (NH_4_)_2_SO_4_ (2.0 g), MgSO_4_ (0.16 g), Na_2_MoO_4_ (0.002 g), CaCl_2_ (0.02 g), FeSO_4_ (0.001 g), MnCl_2_ (0.001 g) and glucose (20 g) per liter of distilled water) with pH of 6.8 (Kumar et al. 2008). The bacterial cells were harvested at log phase (after 48 h of incubation at 37 °C) by centrifugation at 10,000 *g* for 10 min. The cells were further resuspended in 20 ml of minimal buffer (containing K_2_HPO_4_ (10.5 g), KH_2_PO_4_ (4.5 g), (NH_4_)_2_SO_4_ (1 g) and sodium citrate·2H_2_O (0.5 g) per liter). For EMS mutagenesis, 2 ml of diluted suspension of *B. subtilis* was treated with different concentrations of EMS (0.01, 0.02, 0.03, 0.04 EMS in ml) and incubated at 37 °C in an incubator shaker, each for 0, 10, 20, 30 and 40 min. On the other hand, ultraviolet fluorescence analysis (Macros Scientific Works Pvt. Ltd., India) was employed for UV mutagenesis. The UV lamp was about 20 cm above the surface of the cell suspension. During 30 min of the treatment process, 1 ml aliquots were withdrawn and placed on an ice bath for 5 min and then kept in the dark for 30 min. In both cases, all the collected aliquots were then serially diluted up to 10^−6^ and inoculated onto minimal media plates containing 200 μg of 5-bromo-4-chloro-3-indolyl-β-d-glucopyranoside (X-glucoside) with 0.2 % (w/v) cellobiose (minimal media-X-glucoside-cellobiose plates). The plates were incubated at 37 °C for 72 h and LD_50_ was determined. The potential EMS-generated mutant (PS-CM5) was further exposed to UV radiation. Screening and selection of potential mutants were done based on their β-glucosidase production. The positive strains producing β-glucosidase were identified as blue-colored colonies among the negative strains that were white colonies. Colonies were picked from minimal media plate on the basis of their color intensity and size. The selected colonies were further screened for higher enzyme production and genetic stability.

### β-Glucosidase production in parent and mutant strains

The parent strain (PS) and mutants were batch cultured in minimal medium supplemented with 0.1 % glucose and incubated for 72 h at 37 °C in a 120 rpm orbital shaker. β-Glucosidase activity was assayed by incubating the reaction mixture containing 10 mM p-nitrophenyl-β-d-glucopyranoside (pNPG) and culture filtrate in 1:1 ratio for 1 h at 37 °C. The reaction was stopped by adding 2 ml of 1 M Na_2_CO_3_. The p-nitrophenol release was monitored at λ_405 nm_ in UV–Vis spectrophotometer (Jäger et al. 2001). Protein content was determined by the method of Bradford (Bradford 1976). One unit (U) of β-glucosidase was defined as the amount of the enzyme to produce one μmole p-nitrophenol per minute under the assay conditions and the specific activity was defined as the number of units per mg of protein. β-Glucosidase production was calculated as enzyme units produced per liter of media (U/l) (Rajoka et al. 2004). In this step, only the best performing mutants (one each from the three mutagenesis strategies) were selected for further studies.

### Genetic stability of selected mutant strains

The genetic stability of the selected mutants was determined by measuring the levels of β-glucosidase production for successive generations. The interval for each generation was 15 days (Jun et al. 2009).

### Effect of incubation time on β-glucosidase production in parent and mutant strains

The effect of incubation period on β-glucosidase production by parent (PS) and selected mutant strain (PS-CM5-UM3) was studied. The growth and β-glucosidase production of both strains were determined at every interval of 2 h for 36 h at 37 °C.

### Optimization of culture conditions

The culture conditions were optimized by the classical “one-variable-at-a-time approach”. Minimal broth with different pH (3–9) and incubation temperatures (20–70 °C) were studied for 30 h. The bacterial growth and β-glucosidase production were studied up to 30 h of incubation period. The effect of various nitrogen sources on β-glucosidase production was also studied. Organic nitrogen sources (peptone, yeast extract, methionine and glycine) at 1 % concentration and inorganic sources (urea, potassium nitrate and ammonium nitrate) at 0.1 % concentration were added to the minimal media. Culture growth was measured at 600 nm, while β-glucosidase production was measured as enzyme units produced per liter (U/l).

### Partial purification of β-glucosidase

Ammonium sulfate was added to the cell-free culture supernatant up to 75 % saturation and the solutions were kept at 4 °C overnight. The precipitated protein was collected by centrifugation at 10,000 rpm for 20 min at 4 °C. The supernatant was decanted and the pellet was dissolved in minimum amount of acetate buffer (0.1 M, pH 5.0). The protein content and β-glucosidase activity were measured in each fraction. The ammonium sulfate fraction was carefully transferred into the dialysis tubes (10 cm) activated by boiling in solution containing 2 % (w/v) NaHCO_3_ and 1 mM EDTA. This was followed by washing repeatedly with lukewarm water. The ammonium sulfate fractions were dialyzed against 0.1 M acetate buffer (pH 5.0) on magnetic stirrer at 4 °C for 10–12 h with repeated changes of buffer. The partially purified fraction was concentrated against crystals of sucrose. All steps were performed at 4 °C. The fraction obtained was then assayed for protein content and β-glucosidase activity.

### Size-exclusion chromatography with Sephadex-100

The partially purified β-glucosidase was further purified using Sephadex G-100 (size-exclusion limit 4–150 kDa) gel filtration columns (0.5 × 20 cm). The standard molecular weight marker proteins used for the determination of molecular weight were catalase (240 kDa), bovine serum albumin (66 kDa), ovalbumin (43 kDa) and lysozyme (14.3 kDa). 3 ml of the isolated concentrated protein sample (2.8 mg/ml) was applied on the column and eluted with a flow rate of 1 ml per minute. Fractions (1.5 ml) were collected. The protein content and the β-glucosidase activity in each of these fractions were determined by taking the absorbance at λ_280 nm_.

### Analysis of protein samples by SDS (sodium dodecyl sulfate) polyacrylamide gel electrophoresis

The SDS-PAGE of partially purified protein samples was done using the method described by Laemmli (1970) with 10 % separating gel and 5 % stacking gel. After the preparation of the gel, the enzyme obtained after gel-filtration chromatography containing 40 μg of protein was loaded on the gel. The gel was run under a constant voltage of 80 volt till the sample was in stacking gel and after that the voltage was raised to 100 volts. The gel was allowed to run till the dye front reached 0.5 cm from the lower edge of the gel.

### Determination of thermostability

The optimum temperature for the purified β-glucosidase was determined by incubating the reaction mixture at different temperatures; ranging from 30 to 70 °C for 30 min. The β-glucosidase activity was determined as described above and OD was taken at λ_405 nm_. The graph was plotted for temperature versus β-glucosidase activity (% of maximum).

### Statistical analysis

Graphs were plotted either using Origin 6.0 or Microsoft Excel 2003. Each value in graphs and tables represents the mean of triplicate measurement and expressed as mean ± standard error at *p* < 0.05.

## Results and discussion

### Mutagenesis and screening/selection of mutants

Random mutagenesis using chemical agents and UV irradiation is an easy tool to achieve genetic and functional modifications of an organism. For mutagenesis experiments, 50 % lethality dose (LD_50_) for both EMS mutagenesis and UV mutagenesis was determined. It was found to be 0.03 ml EMS exposure for 20 min and 25 min exposure to far UV source from a distance of 20 cm. Chandra et al. (2009) have found that 99 % killing of *Trichoderma reesei* spores was achieved using *N*-methyl-*N*′-nitro-*N*-nitrosoguanidine (NG) for 6 h, followed by UV irradiation for 15 min. Santiago et al. (2006) reported that 50 % lethality was achieved after 3 min UV irradiation of *Aspergillus niger* spore suspension.

On the basis of color intensity and the size of the colonies on X-glucoside-containing plates, eight mutants after EMS mutagenesis, three after UV mutagenesis and nine after EMS plus UV mutagenesis were recovered and screened on the basis of their β-glucosidase production level (Table 1). Three mutants (PS-UM1, PS-CM5 and PS-CM5-UM3) were selected after primary screening for further studies. β-Glucosidase production in mutant PS-CM5-UM3 (806.10 U/l) was found to be better in comparison to other mutants (762.53 U/l for PS-UM1 and 806.10 U/l for PS-CM5, respectively). Therefore, under the same conditions, the mutant PS-CM5-UM3 showed 1.2-fold increase in β-glucosidase production over the parent strain PS. In a similar study, the mutant strain of *B. amyloliquefaciens* gave around 1.4-fold higher alpha amylase activity than the parental strain after mutation with EMS and UV radiations (Haq et al. 2010). Dillon et al. 2006 also reported 1.5-fold more cellulase productivity in *Penicillium echinulatum* by three repeated mutagenic treatment steps of UV, EMS and H_2_O_2_ (Durand et al. 1988). Chandra et al. (2009) have suggested that the enhancement of enzyme activities might be due to increased level of modulator proteins or co-factors promoting the enzyme activities.

**Table 1:** β-glucosidase production in parent strain and screened mutants

Strain/mutant	β-glucosidase production (U/l)
Without mutagenesis	
PS (parent)	675.38 ±28.43
EMS mutagenesis	
PS-CM1	108.93 ± 4.56
PS-CM2	196.08 ± 8.96
PS-CM3	544.66 ± 26.67
PS-CM4	65.36 ± 2.88
PS-CM5	762.53 ± 36.54
PS-CM6	283.22 ± 13.21
PS-CM7	675.38 ± 29.75
PS-CM8	718.95 ± 34.43
UV-mutagenesis	
PS-UM1	718.95 ± 34.08
PS-UM2	65.36 ± 2.98
PS-UM3	457.52 ± 20.45
EMS plus UV-mutagenesis	
PS-CM5-UM1	762.53 ± 38.12
PS-CM5-UM2	283.22 ± 15.02
PS-CM5-UM3	806.10 ± 38.98
PS-CM5-UM4	108.93 ± 5.34
PS-CM5-UM5	631.81 ± 28.60
PS-CM5-UM6	675.38 ± 32.04
PS-CM5-UM7	21.79 ± 1.08
PS-CM5-UM8	65.36 ± 3.42
PS-CM5-UM9	326.80 ±12.74

### Genetic stability of mutants

**Fig. 1:**
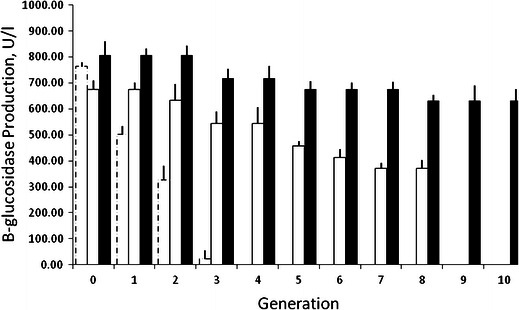
Genetic stability evaluation for the selected mutants. The genetic stability of the desired mutants was determined by measuring the levels of β-glucosidase production up to ten successive generations; the interval for each generation was 15 days. Mutant PS-CM5 (*empty bar with dashed line*); mutant PS-UM1 (*empty bar with solid line*); and mutant, PS-CM5-UM3 (*black*)

The genetic stability of the selected mutants (PS-UM1, PS-CM5 and PS-CM5-UM3) was determined by measuring the β-glucosidase production for ten successive generations. The PS-CM5 mutant strain was unstable in comparison to other selected mutants (PS-UM1 and PS-CM5-UM3). PS-UM1 was stable up to the eighth generation, while PS-CM5-UM3 was stable up to the tenth generation with 78 % of initial β-glucosidase production (Fig. 1). Hence, PS-CM5-UM3 was finally selected and characterized. In a similar report, fungal *T. reesei* mutant was found to be quite stable for cellulases and xylanase production up to seven generations (Jun et al. 2009).

### Growth and β-glucosidase production levels of parent and mutant strains

**Fig. 2:**
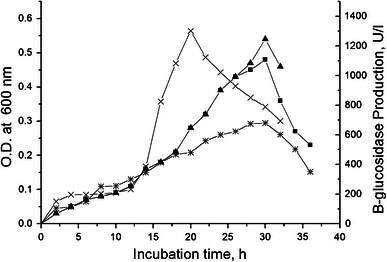
Growth and β-glucosidase production in parent and mutant strain. Both parent and mutant grow in minimal media up to 36 h. Growth was measured at OD_600nm_ and β-glucosidase production was measured by standard method after 2 h interval. Growth of parent strain PS (*filled squares*); growth of mutant PS-CM5-UM3 (*filled triangles*); enzyme production of parent strain, PS (*asterisks*); and enzyme production of mutant PS-CM5-UM3 (*times symbols*)

The selected mutant (PS-CM5-UM3) secreted two times higher β-glucosidase (1,302 U/l) after 20 h of incubation (Fig. 2) in comparison to the parent *B. subtilis* strain PS (680 U/l after 30 h). A similar finding was reported in the reduction of α-amylase production time in *B. amyloliquefaciens* from 72 to 48 h after mutation (Haq et al. 2010).

### Effect of pH and temperature on bacterial growth and β-glucosidase production

**Fig. 3:**
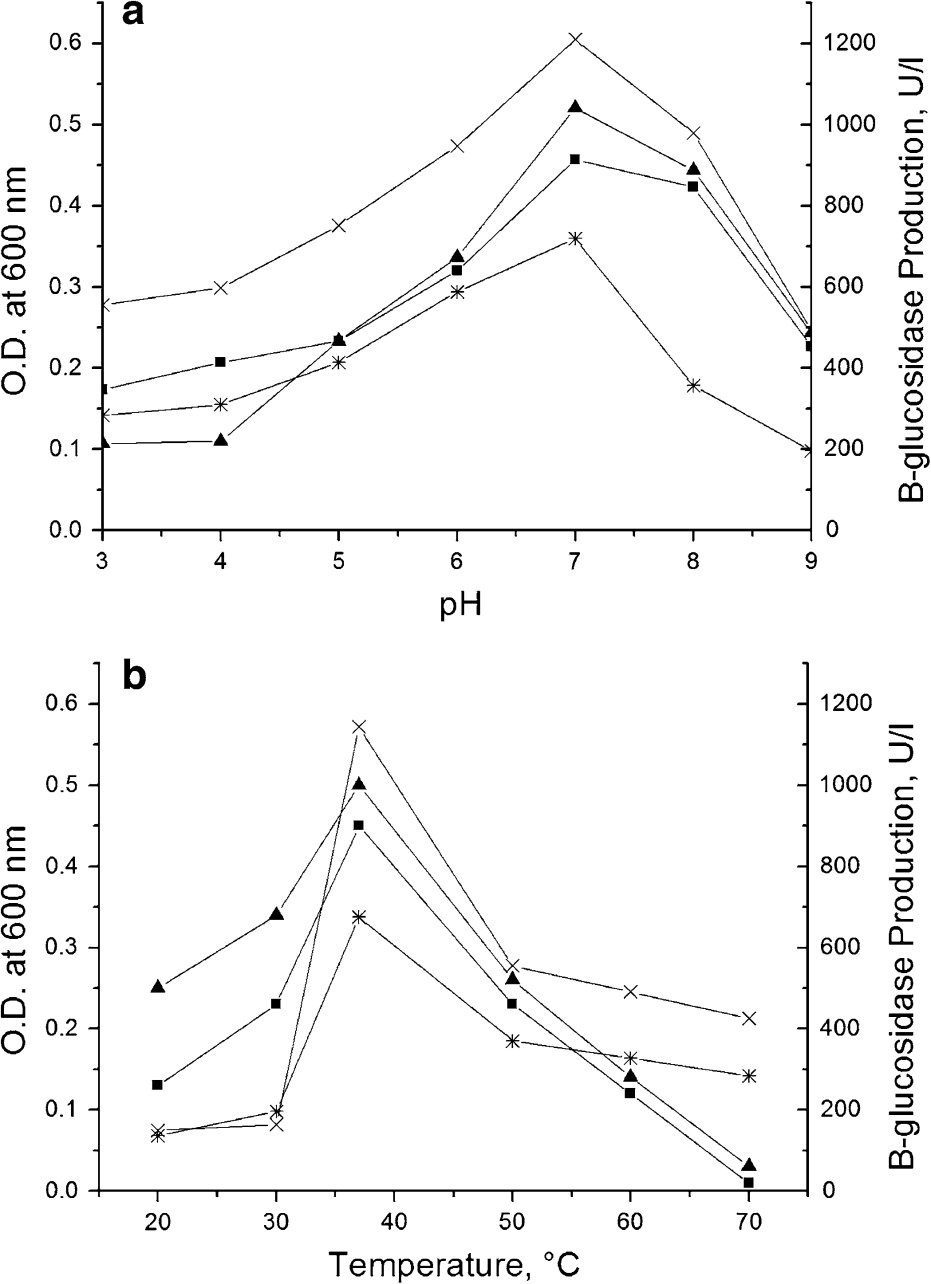
Growth and β-glucosidase production in parent and mutant strain at different pH (**a**) and temperatures (**b**). Growth of parent strain, PS (*filled squares*); growth of mutant strain, PS-CM5-UM3 (*filled triangles*); enzyme production of parent strain, PS (*asterisks*); and enzyme production of mutant strain, PS-CM5-UM3 (*times symbols*) strain of *B. Subtilis*

The maximum β-glucosidase production in parent (PS) and mutant (PS-CM5-UM3) was found at pH 7.0, i.e., 718 U/l and 1,209 U/l, respectively (Fig. 3a, b). Both at lower and higher pH than 7.0, poor growth was observed in both the parent and mutant strains with only meager amounts of β-glucosidase production. Similarly, maximum β-glucosidase production in parent (PS) and mutant (PS-CM5-UM3) was found with incubation at a temperature of 37 °C, i.e., 675 and 1,144 U/l, respectively. Temperatures lower or higher than 37 °C suppressed the growth and β-glucosidase production in both the parent and mutant strains.

### Effect of nitrogen sources on β-glucosidase production

Different nitrogen sources play a critical role in enzyme production. Therefore, the effect of various nitrogen sources on bacterial growth and β-glucosidase production was assessed. In parent strain PS, the maximum β-glucosidase production (1,198 U/l) was achieved when (NH_4_)_2_SO_4_ was used as nitrogen source, while the mutant PS-CM5-UM3 produced maximally (1,797 U/l) when peptone was used as the nitrogen source in minimal media. The β-glucosidase production level of mutant was 1.5 times higher than the parent strain in the presence of peptone (Table 2). PS-CM5-UM3 also showed increased β-glucosidase production (1,536 U/l) with (NH_4_)_2_SO_4_. Recently, Aparna et al. (2012) have reported that the choice of nitrogen source affects the biosurfactant production from *Bacillus clausii* strain. Demirkan (2011) has reported that tryptone was found to be better for amylase production by the parental and mutant strains in comparison to other nitrogen sources. Daroit et al. (2007); Barbosa et al. (2010) and Joo et al. (2010) have indicated that the production level of β-glucosidase was decreased when potassium nitrate was used as the nitrogen source. Aiyer (2004) and Felix et al. (1996) have reported that (NH_4_)_2_HPO_4_ was found to be the best nitrogen source for amylase production. Thippeswamy et al. (2006) have found that among the different nitrogen sources, peptone and yeast extract produced maximum amylase. Hence, the above reports suggest that diverse nitrogen sources may affect the production level of substrates differently. As no clear-cut explanation can be found in the above reports, it is imperative to study the effect of different nitrogen sources.

**Table 2:** Effect of nitrogen source on β-glucosidase production in parent and mutant

Nitrogen source	β-glucosidase production (U/l)	
	Parent strain (PS)	Mutant strain (PS-CM5-UM3)
Control	675.38 ± 26.80	1,339.87 ± 52.48
1 % Peptone	762.53 ± 30.45	1,797.39 ± 53.54
1 % Yeast extract	936.82 ± 39.00	1,666.67 ± 75.08
0.1 % Urea	239.65 ± 9.21	816.99 ± 34.00
0.1 % Potassium nitrate	21.79 ± 0.83	32.68 ± 0.93
0.1 % Ammonium sulfate	1,198.26 ± 42.33	1,535.95 ± 59.32
1 % Methionine	457.52 ± 16.74	359.48 ± 62.54
1 % Glycine	283.22 ± 11.34	163.40 ± 6.76

### Partial purification of β-glucosidase and its characterization

**Fig. 4:**
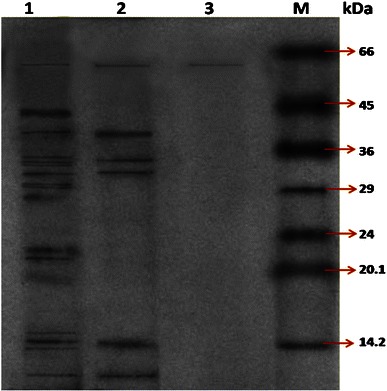
SDS-PAGE of purified β-glucosidase from mutant PS-5CM-UM3: *lane 1*, crude protein; *lane 2*, ammonium sulfate precipitate; *lane 3*, gel filtration chromatography, *M* molecular weight marker of protein standard

β-Glucosidase from a mutant of *Bacillus subtilis* was successfully purified through ammonium sulfate precipitation, Sephadex G-100 and column chromatography (Table 3). β-Glucosidase was purified fivefold with 40 % retention of total extracellular activity. The specific activity of the purified enzyme was 391 U/mg of protein. These results coincide with those obtained by Han and Chen 2008, who obtained a 5.3-fold purification with 44 % recovery after purification through CM Sepharose and 16-fold purification with 21.5 % recovery after purification through Sephadex-75. SDS-PAGE analysis of the purified β-glucosidase indicated the presence of a single band when stained with Coomassie Blue R-250 (Fig. 4). Its apparent molecular mass was about 60 kDa. Han and Chen (2008) reported that β-glucosidase is represented by a single band of Mw 62.4 kDa. Karnchanatat et al. (2007) also reported that, SDS-PAGE analysis of the purified β-glucosidase showed the presence of a single band when stained with Coomassie BlueR-250 and its apparent molecular mass was about 64.2 kDa.

**Table 3:** Partial purification of β-glucosidase

	Total activity (U)	Total protein (mg)	Specific activity (U/mg)	Purification (fold)	Yield (%)
Culture filtrate	1,264.05	28.33	44.61	1	100
Ammonium sulfate precipitation	850.19	10.93	77.761	1.74	67.25
Dialysis	611.92	7.08	86.38	1.93	48.41
Sephadex G-100 (gel filteration)	339.21	0.86	391.08	5.02	39.89

### Thermostability of partially purified β-glucosidase

**Fig. 5:**
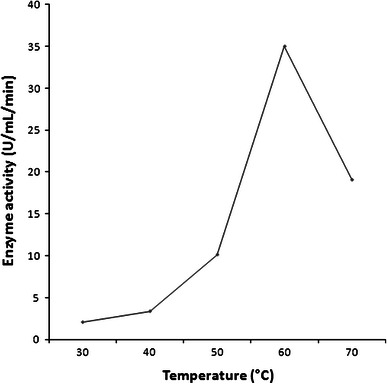
Optimization of incubation temperature for purified β-glucosidase

**Fig. 6:**
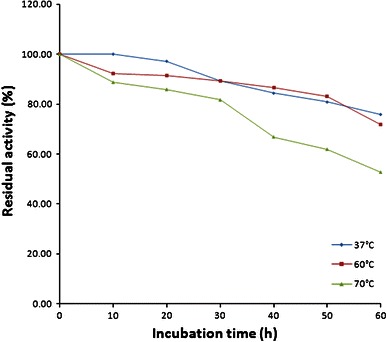
Thermostability of purified β-glucosidase at 37 °C (*filled diamonds*), 60 °C (*filled squares*) and 70 °C (*filled triangles*)

Thermostability of the partially purified β-glucosidase enzyme at different temperatures was monitored. The enzyme displayed maximal activity at 60 °C (Fig. 5) and it was found to be fairly stable at temperatures up to 70 °C for 30 min (Fig. 6). This is in contrast to the β-glucosidases isolated from corn stover which showed maximal activity around 37 °C, and showed depressed activity below 30 °C and above 40 °C. A total loss in activity was observed at 60 °C for 100 min and 70 °C for 1 min (Han and Chen 2008). Temperature stability of β-glucosidase from other sources has been reported to range from 50 to 65 °C (Christakopoulos et al. 1994; Yan and Lin 1997; Yazdi et al. 2003; Karnchanatat et al. 2007).
